# Exploration of physiological sensors, features, and machine learning models for pain intensity estimation

**DOI:** 10.1371/journal.pone.0254108

**Published:** 2021-07-09

**Authors:** Fatemeh Pouromran, Srinivasan Radhakrishnan, Sagar Kamarthi

**Affiliations:** Mechanical and Industrial Engineering, Northeastern University, Boston, Massachusetts, United States of America; Taipei Medical University, TAIWAN

## Abstract

In current clinical settings, typically pain is measured by a patient’s self-reported information. This subjective pain assessment results in suboptimal treatment plans, over-prescription of opioids, and drug-seeking behavior among patients. In the present study, we explored automatic objective pain intensity estimation machine learning models using inputs from physiological sensors. This study uses BioVid Heat Pain Dataset. We extracted features from Electrodermal Activity (EDA), Electrocardiogram (ECG), Electromyogram (EMG) signals collected from study participants subjected to heat pain. We built different machine learning models, including Linear Regression, Support Vector Regression (SVR), Neural Networks and Extreme Gradient Boosting for continuous value pain intensity estimation. Then we identified the physiological sensor, feature set and machine learning model that give the best predictive performance. We found that EDA is the most information-rich sensor for continuous pain intensity prediction. A set of only 3 features from EDA signals using SVR model gave an average performance of 0.93 mean absolute error (MAE) and 1.16 root means square error (RMSE) for the subject-independent model and of 0.92 MAE and 1.13 RMSE for subject-dependent. The MAE achieved with signal-feature-model combination is less than 1 unit on 0 to 4 continues pain scale, which is smaller than the MAE achieved by the methods reported in the literature. These results demonstrate that it is possible to estimate pain intensity of a patient using a computationally inexpensive machine learning model with 3 statistical features from EDA signal which can be collected from a wrist biosensor. This method paves a way to developing a wearable pain measurement device.

## Introduction

Pain assessment is a key step in patient care. Pain is a symptom or sign of numerous medical conditions and is often considered the fifth vital sign. In practice, visual analog scales, numerical rating scales, and verbal rating scales are commonly employed pain assessment measures [1]. However, all these measures come from the patient’s self-reported information, which is subjective in nature. Often this information is not reliable because patients generate their self-reports when they are distracted or impaired by pain, affected by medication, and communication-challenged by intense pain. Moreover, these subjective pain reports, being point-in-time measurements, are not tenable for continuous pain monitoring. Hence, objective measurement of pain has long been clinicians’ goal for effective pain management and treatment.

It is reported that 25.3 million Americans suffer from daily pain, and 23.4 million Americans experience severe pain [2]. It is estimated that the national cost of pain in the US ranges from $560 to $635 billion a year, which is larger than the annual costs for cancer, heart disease, or diabetes and about 30% higher than the combined cost of cancer and diabetes [3]. The prescribed opioid for pain relief must match the patient’s pain intensity. Any over-prescription can lead to opioid dependency, drug-seeking behavior, and harmful consequences such as over-sedation-related deaths. On the other hand, under-prescription of opioids when the patient is really in severe pain can cause physical and mental suffering and patient dissatisfaction [4]. The U.S. Department of Health and Human Services recommended better management of pain as one of the five key strategies to address the opioid crisis, which was declared a nationwide public health emergency in 2017. Misuse of opioids has been found in over 50% of chronic pain patients [5]. The objective pain assessment system can significantly contribute to preventing drug abuse by providing decision support to prescribe the pain medication comparable to pain intensity.

In this paper, we explored different automated objective pain estimation models built on easy to measure physiological sensors and effective signal features.

## Related works

Automated pain assessment is an evolving research area that has received increasing interest among machine learning researchers. However, most of the studies have focused on models that use images and videos monitoring facial expression and body movement as behavioral indicators of pain [6–8]. These expression-driven methods have some limitations that make them less practical in a real-world clinical setting. First, recording videos of patients can create privacy concerns and make patients self-conscious. Second, images and video recording require careful and involved setup to adjust for lighting conditions and to keep the focus on moving patients. Third, patients can easily exaggerate their pain intensity by affecting pain in their facial expressions to cater to their drug-seeking behavior. Fourth, video-based methods do not lend themselves for including in wearable devices synched to smartphones. For these reasons, researchers have shifted focus on investigating physiological pain biomarkers.

The main idea of using the physiological signals for pain estimation comes from the autonomic nervous system’s role in pain response. The pain process often starts with the activation of the sensory neural pathway by noxious mechanical, heat, cold, chemical, or inflammatory stimuli which is applied to the body from an external or internal source. The information regarding damage impact is transduced through neural pathways and transmitted through the peripheral nervous system to the central and autonomic nervous systems. Then, the signal is sent to higher levels from the spinal cord to the centers of the prosencephalon, mesencephalon, and cortex in the brain, resulting in pain perception [9–11]. In this process, activation of the autonomic nervous system triggers physiological alterations in the skin, heart, and muscles’ electrical properties. This physiological response to pain makes EDA, ECG, and EMG signals potentially good candidates for developing a machine learning model for objective pain measurement.

Electrodermal Activity (EDA), which is also referred to as Skin Conductance (SC) or Galvanic Skin Response (GSR), measures the changes in the electrical properties of the skin. In response to a pain stimulus, the sympathetic nervous system innervates sweat glands to secrete sweat. This emotional sweating decreases the skin’s electrical resistance, which is equivalent to an increase in the skin’s conductivity until the sweat is reabsorbed or evaporated [12–14]. EDA signal has drawn the attention of many researchers due to its portability and ease of collection. Momin et al. [15] have recently reviewed the EDA signal’s essential signal characteristics and theoretical, physiological and psychological significance. EDA signal is composed of phasic and tonic components which measure the short-term variation and long-term variation of the signal, respectively. Lopez-Martinez et al. [16] proposed deconvolving the EDA signal into its tonic and phasic components to obtain measures that better represent the sympathetic nerve activity in response to pain. Sympathetic nervous system reactivity to nociceptive stimulation also leads to changes in time and frequency of heartbeats [17]. ECG records the electrical activity of the heart during heartbeats. Acute pain increases the heart rate, changes the heart rate variability, and significantly increases the low-frequency power. EMG records the electrical activity of muscles. EMG for trapezius muscle is considered an indicator of sympathetic and parasympathetic nervous system activation during physiological arousal in response to pain [18]. In low back pain (LBP) patients, EMG has been used as an information carrier for diagnosis and treatment. EMG signal is a noninvasive measurement technique but often inconvenient and hard to use in a real-world setting. Rodríguez-Tapia et al. [19] and Campanini et al. [20] recently reviewed the clinical value of EMG signal, proposed guidance for signal processing, and addressed barriers to its application in real-world settings.

Different studies have focused on exploring a relationship between pain and physiological signals such as electrodermal activity, heart rate variability, brain activity, blood pressure, and respiration rate. Most of them have only focused on pain recognition, which means that they just indicated statistical significance correlations between individual physiological signals and pain to recognize the presence or absence of pain, without quantifying the pain intensity. Naranjo-Hernández et al. [21] provided a detailed review of different sensors that have been proposed for objective evaluation of chronic pain.

There are a growing number of studies investigated physiological signals and machine learning models for objective pain intensity estimation, but they have not yielded estimation accuracies acceptable in clinical settings. Therefore, there is a need for developing a high-performance model to build a reliable pain assessment device practical for clinical and personal use. Chu et al. [22] used linear discriminant analysis (LDA on BVP, ECG, and EDA signals of six healthy subjects aged between 22 and 25 years old to classify the subjects’ pain into five different pain intensities induced by electrical stimulation. In another study [23], they also built support vector machines (SVMs) and k-nearest neighbors (KNN) models on the same dataset. Walter et al. [24] used EDA, ECG, and EMG signals from the BioVid Heat Pain dataset to build SVM models to classify each of the four levels of pain intensity against no pain. They extracted features from mathematical groups of amplitude, frequency, stationary, entropy, linearity and variability. Gruss et al. [25] extended this work by adding a set of similarity features. Kachele et al. [26] built random forest classifiers using EDA, ECG and EMG signals from the same BioVid dataset, together with meta-information and similarity. Most of these studies focus on binary classification between no pain and a specific level of pain. Kachele et al. [26] advanced pain assessment task from classification to estimation. They treated pain intensity as a continuous variable instead of as an ordinal variable with fixed categories. They developed a regression model that is less restrictive and more practical for pain monitoring. Kachele et al. [27] presented the first regression model for continuous pain estimation using an Adaptive Random Forest model and EDA, ECG, EMG and video stream features. Lopez-Martinez et al. [28] also presented a recurrent neural network model using EDA signal for continuous pain intensity estimation. An extensive review of automatic pain assessment methods can be found in Werner et al. [11] and Wagemakers et al. [29].

With the success of deep learning methods in various domains, researchers have recently investigated their application in pain recognition. Thiam et al. [30] explored deep learning models for pain classification. They showed improvement in the binary classification between no pain level and the highest pain level, using 1D convolutional neural networks on raw EDA signal with minimum preprocessing. In their subsequent work [31], they proposed a multi-modal information fusion approach based on deep denoising convolutional autoencoders for the binary classification task on EDA, EMG and ECG assessed on BioVid dataset. Yu et al. [32] proposed the diverse frequency bandpass-based CNNs for subject-dependent tonic cold pain assessment using EEG signal. They first extracted various feature representations from different frequency bands of the 32-channel EEG signal. These features were then concatenated and fed into a fully connected network that classified pain states with three tonic pain levels. Wang et al. [33] proposed a BiLSTM RNN model to learn the temporal dynamics of the BioVid dataset’s physiological signals and then fused these RNN-generated features with a set of hand-crafted features for binary pain classification tasks. Recently, Subramaniam and Dass [34] proposed a multi-modal hybrid CNN-LSTM model for binary pain classification using EDA and ECG signal from the BioVid dataset.

Automated pain assessment is complex due to its subjectiveness and inter-subject variability in autonomic nervous system reactivity. Significant variability among people in their pain perception and physiological responses to pain is one of the challenges in developing a general pain recognition model. Even in the same individual, pain responses can be influenced by emotional states and other environmental conditions. On the other hand, collecting a large sample set for each new subject and rebuilding a machine learning model based on that data seems less practical in real-world settings. To address this problem, different studies [35–37] investigated the use of a multi-task-learning approach for personalized pain recognition from physiological signals in which a model is basically trained on the general population data but explicitly tailored for individuals. They showed that this approach could provide pain detection with better performance. In [38], Lopez-Martinez et al. proposed an algorithm that employs RNNs to automatically estimate pain intensity levels from face images and then fed it into the personalized Hidden Conditional Random Fields to estimate VAS for each person. In [39], the authors derived a personalization approach for pain detection using Bayesian hierarchical modeling with wavelet features from functional near-infrared spectroscopy (fNIRS) signals.

Machine learning models built on physiological signals have already shown promising results in domains such as automated emotion recognition, stress detection, health monitoring, and disease diagnosis [40–43]. Ihmig et al. [44] explored machine learning algorithms and combination of features from ECG, EDA and respiration (RSP) signals for two-level and three-level classification of anxiety level. Kim et al [45] demonstrated that selected set of features from EDA signal can be used as a good biomarker for automatic detection of major depressive disorder using a decision tree classifier. Pineda et al. [46] presented a machine learning model for diagnosis of Alzheimer’s disease using EEG signal. They extracted five typological network features from quantile graphs of EEG signal and presented them to SVM classifier to detect the disease using data collected from 24 healthy and 24 patient subjects. It has also been demonstrated that ECG-based SVM model have the potential to accurately detect Atrial Fibrillation arrhythmia using wearable technology to prevent AF-related stroke [47]. Moreover, studies have also proven that Biosignal-based Machine learning models are useful for automated sleep monitoring and sleep stage classification [48,49].

## Materials and methods

### Data source and experiment procedure

The data used in this study was not generated by our research group. We used the data from the publicly available BioVid Heat Pain Database Part A [24]. We obtained this data by contacting the BioVid research team lead, Philipp Werner [50], through biovid-db@ovgu.de. The readers can obtain the data through the same process. The authors had no special access privileges to the data. The data includes 87 healthy participants in the age range of 18–65, approximately balanced across genders. The experimental setup employed thermode (PATHWAY, http://www.medoc-web.com) to induce heat pain in the right arm. The experiment considered 32°C as the baseline temperature. For each participant, they found temperature (*T*_1_) at which the subject starts to experience pain and the temperature (*T*_4_) at which the subject cannot tolerate the pain anymore. Then they added two intermediate temperature levels (*T*_2_ and *T*_3_) such that *T*_1,_ *T*_2,_ *T*_3,_ and *T*_4_ are consecutively separated by an equal distance. Thus, there were four subject-dependent temperatures to induce four pain levels, 1 to 4; these pain levels are labeled as P_1_, P_2_, P_3_ and P_4_ respectively. In the 25-minute-long experiment, a subject was made to experience a mix of the four temperature intensities in random order, each temperature appearing in total 20 times in the mix. The subject experienced each temperature level (i.e. each pain level) for 5.5 seconds followed by a no-pain recovery phase at the baseline temperature of 32°Cfor 8–12 seconds. No-pain state, labeled pain level 0 (i.e. *P*_0_), is defined as the pain intensity the subject experiences at the last 5.5 seconds of recovery phase ensuing the pain level 1 (i.e. *P*_1_). For each subject, the dataset has 20 records of each of the five pain states, *P*_0_, *P*_1_, *P*_2_, *P*_3_ and *P*_4_. In this dataset, the following sensor signals were recorded: EDA, ECG, and EMG. The sampling resolution of each signal is 512 Hz. In total, the data has 8700 labeled samples for each signal. EMG signal were preprocessed by applying Butterworth band-pass filter with 0.1–250 Hz frequency band through Empirical Mode Decomposition technique. ECG signals were also filtered by Butterworth band-pass filter in frequency range 0.1–250 Hz.

### Feature extraction

Although the relevant features from physiological signals can be extracted manually using pre-defined patterns described in medical literature, we tried to automate the feature extraction process by simply considering the physiological signals as time-series data. We extracted 22 time-series features reported to be most effective by Lubba et al. [51]. They selected these 22 features by exploring about 5000 candidate features for their classification performance on 92 different time-series datasets.

This set of 22 features captures properties including linear and non-linear autocorrelation, successive differences, value distributions and outliers, and fluctuation scaling properties from time and frequency domains of each signal. The description of these selected features is presented in Table 1.

**Table 1:** Description of features extracted from each physiological signal as time-series data.

Feature Category	Feature Description
Distribution	• Mode of z-scored distribution (5-bin histogram) • Mode of z-scored distribution (10-bin histogram)
Simple temporal statistics	• Longest period of consecutive values above the mean • Time intervals between successive extreme events above the mean • Time intervals between successive extreme events below the mean
Linear autocorrelation	• First 1/e crossing of the autocorrelation function • First minimum of the autocorrelation function • Total power in the lowest fifth of frequencies in the Fourier power spectrum • Centroid of the Fourier power spectrum • Mean error from a rolling 3-sample mean forecasting
Nonlinear autocorrelation	Time-reversibility statistic, ((x_t+1 –_ x_t_)^3^)_t_ • Automutual information, m = 2, τ = 5 • First minimum of the automutual information function
Successive differences	• Proportion of successive differences exceeding 0.04σ • Longest period of successive incremental decreases • Shannon entropy of two successive 3-letter symbolization • Change in correlation length after iterative differencing • Exponential fit to successive distances in 2-d embedding space
Fluctuation analysis	• Proportion of slower timescale fluctuations that scale with DFA (50% sampling) • Proportion of slower timescale fluctuations that scale with linearly rescaled range fits
Others	• Trace of covariance of transition matrix between symbols in 3-letter alphabet • Periodicity measure of Wang et al. [52]

### Machine learning models

For automated estimation of pain intensity using physiological signals, we applied different machine learning algorithms on the features extracted from EDA, ECG, and EMG signals to compare their performances and find the best combination of signal, feature set, and machine learning model. For each subject, features were standardized by the subject’s own data to have zero-mean and unit-variance to account for the variations across subjects. First, we built Linear Regression model as a baseline. Then, we applied Support Vector Regression (SVR) which is a powerful kernel-based machine learning algorithm [53]. We utilized Radial Basis Function (RBF) nonlinear kernel as it outperformed other kernel functions such as linear and polynomial kernels in this study. Moreover, we tried Extreme Gradient Boosting Regression (XGBoost) algorithm [54], which is an ensemble technique where in new models are added sequentially to correct the errors in previous learning trees. It uses the gradient descent algorithm to optimize the loss function. We also explored Random Forest and K-nearest neighbor (KNN) algorithms. We used Scikit-Learn [55] and XGboost [54] in Python to build and tune the models. Then we built fully connected neural networks with Tensorflow and Keras [56]. We tuned the model hyperparameters to improve their performance. We used 15% of the samples outside the train and test sets for hyperparameter tuning. We applied the exhaustive grid search approach to adjust the hyperparameters which help us find the balance between bias and variance and thus, prevent the model from overfitting and underfitting. The hyperparameters we explored in GridSearch for each algorithm are presented in Table 2.

**Table 2:** Hyperparameter tuning space for each machine learning algorithm.

ML Algorithm	Parameter	Values/Range
Support Vector Regressor	C	[1, 10, 100, 1000]
	Gamma	[0.01, 0.1, 1, 10, 100]
XGBoost	Min child weight	[1, 5, 10]
	Gamma	[0.5, 1, 1.5, 2, 5]
	Subsample	[0.6, 0.8, 1.0]
	Col. sample by tree	[0.6, 0.8, 1.0]
	Max depth	[3, 4, 5]
KNN	Number of neighbors	[5, 15, 25]
Neural Networks	Number of hidden layers	[1, 2, 3]
	Number of nodes	[32, 64, 128]
	Number of epochs	[10, 20, 30]
	Batch size	[10, 100, 200, 300]

We developed the models based on two pain estimation scenarios defined in the literature [57]. First, we developed a subject-independent model, which is a generic model for all subjects. In this case, the model was built on samples from subjects in the training dataset, and then scored on samples from subjects in the testing dataset. We applied leave-one-person-out cross-validation (LOOCV) in this scenario; we estimated the pain intensity of a new subject based on the patterns discovered on the labeled data samples of other subjects. In the second scenario, we developed a subject-dependent model dedicated to each person individually. The model is both trained and tested on different data samples of the same person. We used a repeated 10-fold stratified cross-validation method for each person in the subject-dependent scenario.

To evaluate the performance of regressor models, we used Mean Absolute Errors (MAE) and Root Mean Square Errors (RMSE) as performance metrics. Given below the definitions of the performance metrics,

$$MAE=\frac{\sum_{k=1}^{n}\left|y-ŷ\right|}{n}\;and\;RMSE=\sqrt{\frac{\sum_{k=1}^{n}{\left(y-ŷ\right)}^{2}}{n}},$$

where *y* is true value, *ŷ* is predicted value and *n* is the number of samples.

## Results and discussion

We discussed the result of subject-independent and subject-dependent models in this section.

### Subject-independent model (generic model)

We used the leave-one-person-out setting and averaged the results over 87 subjects in the study. The response variable is pain intensity, which is a continuous value from 0 to 4, inclusive. Here the objective is to estimate the pain intensity of a new patient with minimum error.

First, we wanted to compare the performance of EDA, ECG and EMG sensors for continuous pain intensity estimation. We built Linear Regression, Support Vector Regression, Neural Networks and Extreme Gradient Boosting models on the features extracted from signals of each sensor type. Table 3 presents the pain intensity prediction performances of each sensor with these models. The performance metrics are Mean Absolute Error (MAE) and Root Mean Squared Error (RMSE) averaged over all subjects. The results indicate that EDA performs significantly better than ECG and EMG modalities for generic pain estimation model (*p*-value < 0.01, *t*-test). We also investigated the performance of combinations of all sensors. We found that early fusion of all physiological sensors does not fare any better results than those given by EDA sensor alone.

**Table 3:** Performance values (Mean ± Standard deviation) of generic pain estimation model for different sensor and model combinations across all subjects.

*Sensor*	*Model*	*Performance*	
		*MAE*	*RMSE*
EDA	Linear Regression	0.99 ± 0.15	1.18 ± 0.18
	SVR	**0.93** ± **0.19**	1.15 ± 0.21
	Neural Networks	0.95 ± 0.17	1.15 ± 0.19
	Random Forest	0.96 ± 0.18	1.15 ± 0.20
	KNN	0.98 ± 0.16	1.17 ± 0.18
	XGBoost	0.95 ± 0.17	**1.13** ± **0.19**
ECG	Linear Regression	1.16 ± 0.10	1.35 ± 0.12
	SVR	1.15 ± 0.16	1.36 ± 0.18
	Neural Networks	1.17 ± 0.11	1.37 ± 0.12
	Random Forest	1.17 ± 0.12	1.36 ± 0.13
	KNN	1.19 ± 0.09	1.39 ± 0.11
	XGBoost	1.16 ± 0.10	1.34 ± 0.12
EMG	Linear Regression	1.20 ± 0.05	1.39 ± 0.06
	SVR	1.20 ± 0.00	1.41 ± 0.00
	Neural Networks	1.22 ± 0.05	1.41 ± 0.06
	Random Forest	1.21 ± 0.05	1.40 ± 0.06
	KNN	1.23 ± 0.05	1.43 ± 0.06
	XGBoost	1.20 ± 0.04	1.39 ± 0.05
Early fusion of EDA+ECG+ EMG	Linear Regression	0.98 ± 0.17	1.17 ± 0.19
	SVR	0.96 ± 0.19	1.16 ± 0.21
	Neural Networks	1.01 ± 018	1.22 ± 0.19
	Random Forest	0.95 ± 0.18	1.14 ± 0.19
	KNN	1.02 ± 0.15	1.21 ± 0.17
	XGBoost	**0.94** ± **0.18**	**1.13** ± **0.20**

As reported in Table 3, we obtained the best MAE = 0.93 from SVR with EDA input. This indicates that on average, the predicted pain intensity differs from the real pain intensity by less than 1 unit of pain on 0–4 scale. This is an important result because when clinicians desire to estimate the pain intensity of a person objectively, rather than rely on the patients’ self-reported information, we can do that just using the EDA signal measurements and be confident that the estimation will not be too far from the actual pain intensity, say by not more than one unit. This ability to estimate objective pain can make a considerable impact on the dosage of prescribed opioids. The feature set identified in this work allows us to use SVR model instead of complicated classification models usually reported in the pain assessment papers. Since the low accuracy of results for multi-class pain classification problems is discouraging, researchers turned their efforts on investigating the performance of 2-class problems, in which the pain is classified into "no pain" and "one specific pain intensity". The best binary classification performance among them is obtained when detecting whether the patient is suffering from the highest level of pain or the patient has no pain at all. However, in a clinical setting, this kind of binary classification task is not as useful as the knowledge of the exact level of pain.

The classification accuracy in the multi-class problem also ignores the fact that pain levels are ordinal, and different misclassifications have different impacts. For example, misclassification of *P*_4_ as *P*_3_ has less clinical impact than misclassification of *P*_4_ as *P*_0_. However, in the classification problems presented in the literature, different misclassifications are treated on par. This problem can be solved by moving from classification to regression of pain using a performance metric such as MAE. In this case, the error between *P*_4_ and *P*_3_ is 1, but the error between *P*_4_ and *P*_0_ is 4. Moreover, by using a metric like RMSE, the larger differences between actual pain and predicted pain are magnified over the smaller differences which is a desirable property in pain estimation for clinical use.

**Fig 1:**
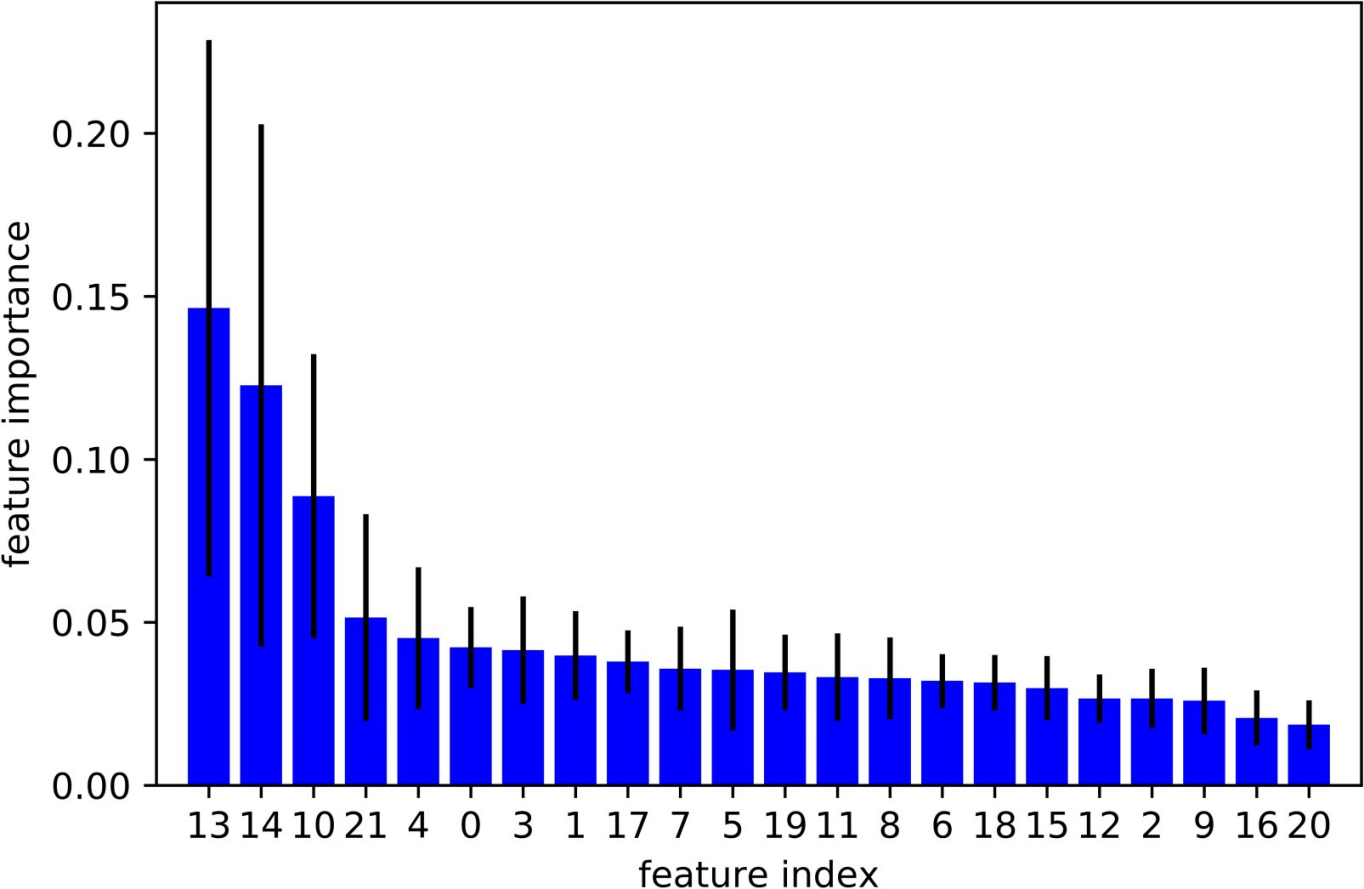
The importance of each EDA feature using Random Forest feature ranking.

**Fig 2:**
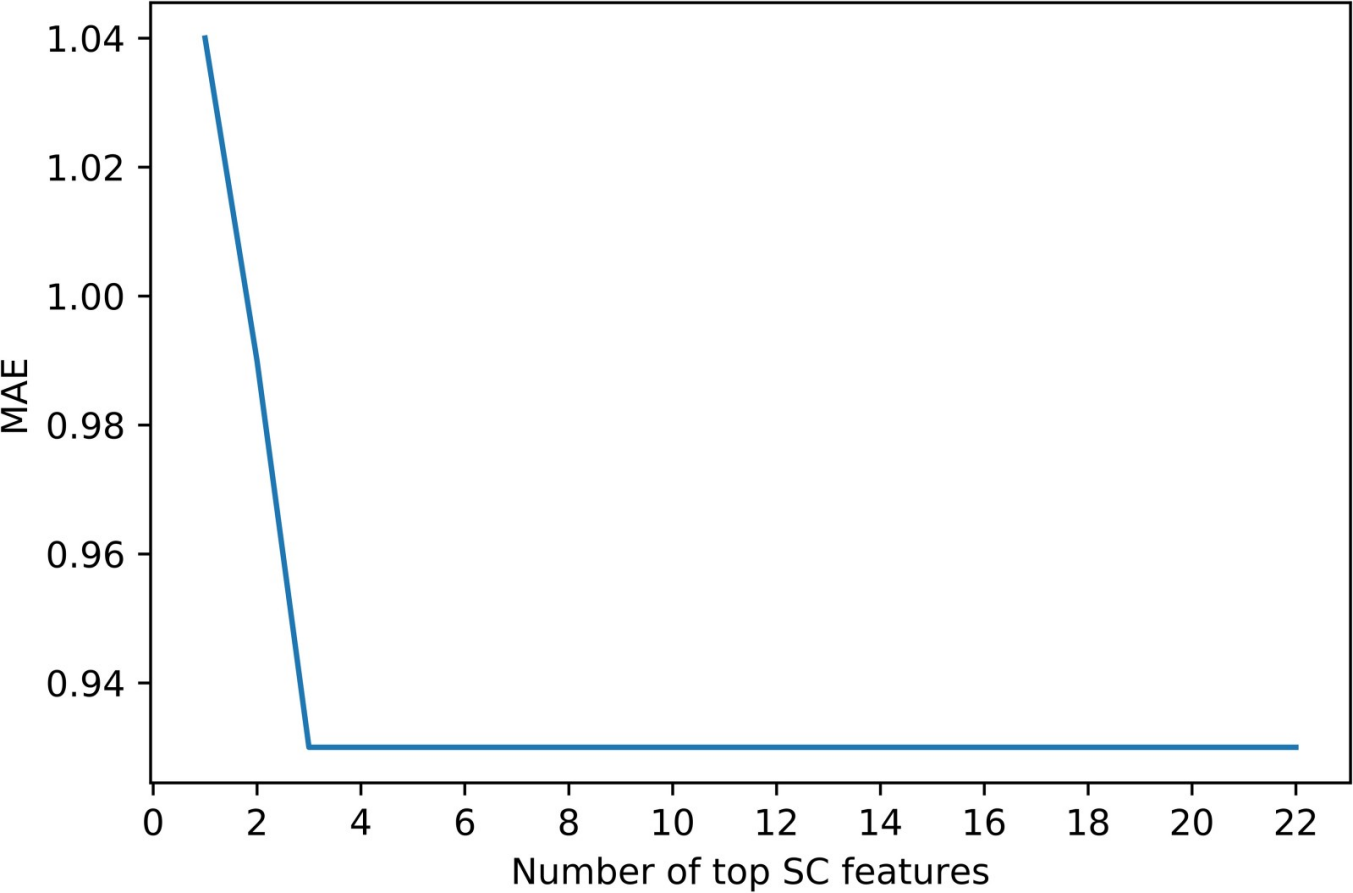
The optimal number of features to be selected.

Although we obtained good results using 22 EDA features, we further explored to see if we can reach similar or better results with fewer EDA features. We conducted a tree-based feature selection exercise to evaluate EDA features on regression trees. We applied feature selection on 10 randomly selected subjects. Each blue bar in Fig 1 indicates the feature importance in the forest of trees, along with their inter-tree variability. This Random Forest feature ranking is computed on the impurity-based feature importance in the forest. Then we used this feature ranking to find out an optimal number of features required to achieve similar or better results. The results are shown in Fig 2. This plot highlights that by considering only the top 3 features, we obtained the same level of performance given by all 22features together. We also explored the XGBoost feature selection method and found that the top 3 EDA features remained same by ranked on the number of times a feature appears in a tree, and gain which is the average gain of splits which use the feature.

The top 3 most informative EDA features are:

- Time interval between successive extreme events above the mean
- Time intervals between successive extreme events below the mean
- Exponential fit to successive distances in 2-dimensional embedding space

**Fig 3:**
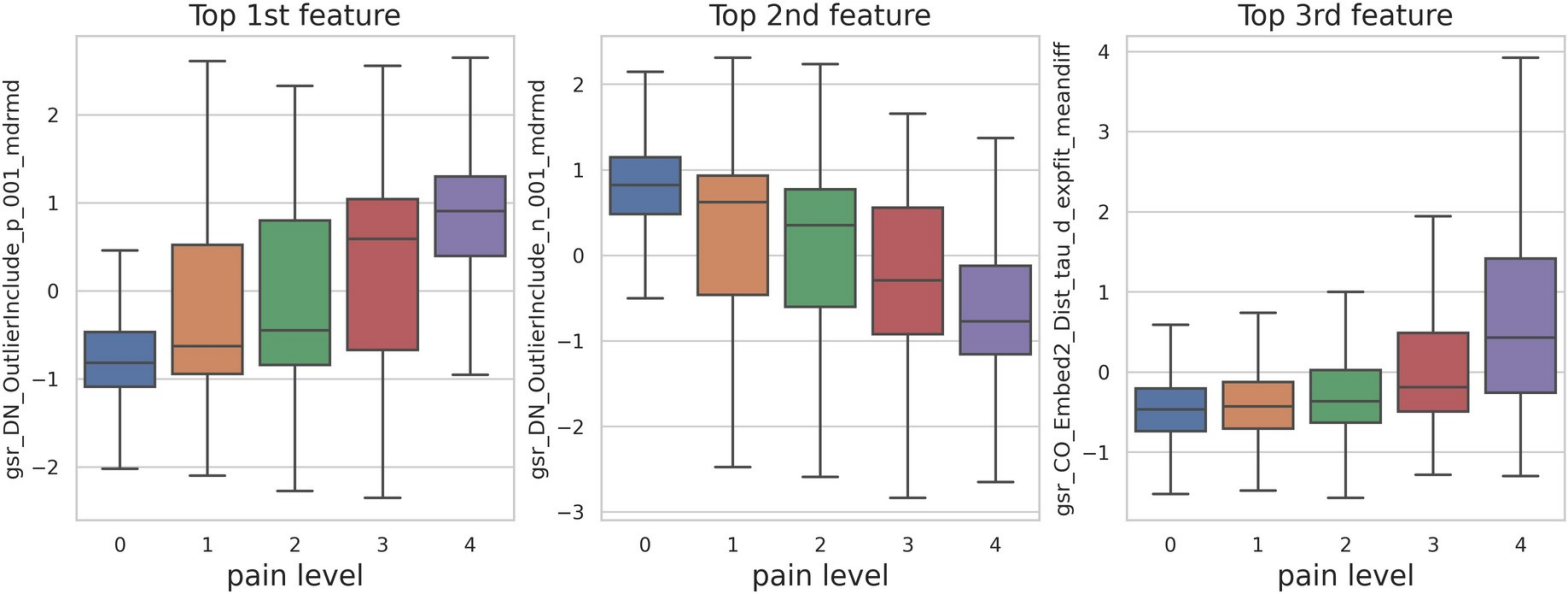
Top 3 EDA features per pain levels.

These features are captured using simple temporal statistics from the distribution of values in the time-series and successive differences. Surprisingly, we can see that when we used these 3 EDA features and ignored the remaining 19 features, the model performance remains the same. We achieved 0.93 (std = 0.21) MAE and 1.16 (std = 0.23) RMSE by using SVR on 3 key EDA features. Likewise, XGBoost gave us 0.96 (std = 0.17) MAE and 0.96 (std = 0.17) RMSE. The MAE of 3-EDA feature model is still significantly below 1-unit error (p-value < 0.05). Fig 3 shows the variation in Top 3 features per different pain levels.

In Table 4, we compared our results with those reported in the literature on the same dataset. Kachele et al. [27] achieved 1.01 MAE and 1.20 RMSE using EDA signal and 0.99 MAE and 1.16 RMSE using the fusion of all the physiological signals and video images. In another study, Lopez-Martinez et al. [28] achieved the best performance of 1.05 MAE and 1.29 RMSE using EDA signal and LSTM recurrent neural network algorithm.

**Table 4:** Comparing the subject-independent pain prediction performances in the literature.

*Study*	*Sensor*	*Model*	*Performance*	
			*MAE*	*RMSE*
Kächele et al. [27]	EDA	Random Forest	1.01 ± 016	1.20 ± 0.18
Kächele et al. [27]	Early Fusion of EDA, ECG, EMG, and Video	Random Forest	0.99 ± 0.17	1.16 ± 0.18
Lopez-Martinez and Picard [28]	EDA	LSTM RNNs	1.05 ± 0.15	1.29 ± 0.16
Current study	EDA	SVR	**0.93 ± 0.21**	**1.16 ± 0.23**

Kächele et al. [27] extracted a large set of EDA signal features including statistical features such as the skewness, kurtosis, ratio between maximum and minimum, number of Skin Conductance response (SCR) occurrences, mean amplitudes of those occurrences, temporal slope of the signal, spectral entropy, and normalized length density. They also used the number of Skin Conductance Level (SCL) responses within a window, the response latency of the first significant response, the average and the area of the phasic driver, the maximum phasic activity, and the mean of the tonic activity. Additionally, the global mean, maximum positive deflection, number of SCL responses and sum of the SCL amplitudes of significant SCL responses were used for pain intensity estimation. Lopez-Martinez and Picard [28] decomposed the EDA signals into phasic and tonic components and then calculated the following features: (1) number of SCRs with onset in the window, (2) sum of amplitudes of all deconvolved SCRs (phasic) with onset in the window, (3) average phasic driver activity, (4) maximum phasic driver activity, (5) integrated phasic driver activity, and (6) mean tonic activity.

Table 4 shows that the pain prediction performance we achieved in this work significantly better than the results reported in the literature on the same BioVid dataset (*p*-value < 0.005, *t*-test). The results also indicate the effectiveness of the 3-EDA feature set that could outperform the results given by the complex algorithms such as RNN. Table 4 also indicates that SVR model with Radial Basis Function (RBF) kernel using just 3 EDA features resulted in a smaller MAE (p-value <0.05, t-test) than the reported result in the literature when they used thousands of features from all physiological signals and videos.

To the best of authors knowledge, there are no other studies on the BioVid dataset that explored the pain intensity estimation as a continuous regression task, excepting the ones listed in Table 4. Other studies performed pair-wise binary classification tasks between baseline and pain state. Although it is not the aim of this study, we also performed the binary classification task between Baseline (P0) and highest pain level (P04) and compared it to the two most recent studies on this dataset. As can be seen in Table 5, our proposed feature set demonstrated a competitive pain detection model performance. While features in the competing models are unexplainable, our signal features are statistical and intelligible.

**Table 5:** Comparing P0 vs P04 subject-independent binary classification task in the literature.

*Study*	*Sensor*	*Model*	*Accuracy (%)*
Wang et al. [33]	EDA+ECG+EMG	RNN-ANN	83.3
Subramaniam et al. [34]	EDA	CNN-LSTM	80.1
Current study	EDA	SVM	83.3

### Subject-dependent model

In addition to generic models, we explored subject-dependent models which are custom trained for individual subjects using subject’s own data. We explored subject-dependent models in which we predicted the pain intensity of a person using the models training only on the person’s own data. This scenario is useful for developing smart personalized devices based on each patient’s own data. We explored two well-performing algorithms, SVR and XGBoost, on each physiological signal. As presented in Table 6, EDA remains the best individual signal for pain estimation in a subject-dependent scenario; it gave an average MAE of 0.93 and an average RMSE of 1.13. Early fusion of all signals again did not significantly improve the predictive performance compared to the performance given by the lone EDA sensor. When we compare each physiological sensor across generic and subject-dependent scenarios, we see that EDA sensor performed almost at the same level in both the cases. However, ECG and EMG gave better results in subject-dependent models than in generic models.

**Table 6:** The pain estimation performance of subject-dependent model for different sensors and models (Mean ± Standard deviation).

*Sensor*	*Model*	*Performance*	
		*MAE*	*RMSE*
EDA	SVR	**0.93** ± **0.24**	**1.13** ± **0.27**
	XGBoost	0.94 ± 0.27	1.13 ± 0.30
ECG	SVR	1.07 ± 0.20	1.27 ± 0.23
	XGBoost	1.08 ± 0.24	1.28 ± 0.27
EMG	SVR	1.13 ± 0.14	1.34 ± 0.15
	XGBoost	1.17 ± 0.20	1.38 ± 0.22
Early fusion of EDA+ECG+EMG	SVR	0.93 ± 0.24	1.12 ± 0.27
	XGBoost	0.91 ± 0.27	1.10 ± 0.30

Hence, after tuning the parameters of both SVR and XGBoost, we achieved less than a 1-unit error for continuous pain estimation in the subject-dependent scenario with only 3 features from the EDA signal of the subject. As presented in Table 7, both SVR and XGBoost models performed equally well for pain estimation in this scenario.

**Table 7:** Subject-dependent model performance with top 3 EDA features.

Signal Features	Model	MAE	RMSE
EDA with top 3 features	SVR	0.92 ± 0.26	1.13 ± 0.28
EDA with top 3 features	XGBoost	0.93 ± 025	1.12 ± 0.28

### Personalization vs. generalization

Implementing a person-specific model which relies only on each subjects’s own data has some challenges in real-world setting. We can only capture a limited number of labeled samples for each new person practically. To address this problem, it is preferable to use a machine learning model that leverage data from the overall population but can be fine-tuned to serve as a subject-dependent model. In this case, it may be a good idea to have a hybrid model combining subject-dependent and subject-independent approaches to benefit from the advantages of both approaches. Transfer-learning approaches like Multi-task Neural Networks or simple personalized fine-tuning on a Multi-modal generic Neural Network model have shown to be beneficial in other studies. In this study, we explored the clustering-based-SVR model and evaluated the model performance on each physiological signal separately to see the value of our combined approach on each signal.

First, we clustered the population data and built cluster-specific models and compared their performance with that of a generic model trained on population data. In this approach, when a new patient comes in, we position the patient in a closest cluster using his/her 22 signal features, and then we use the cluster-specific model to assess the pain intensity of the patient. Hence, this approach seems less challenging than person-specific approach because we don’t need to do a signal recording for a long period of time, and we don’t need to build a new model whenever a new patient comes in. In this model, we hypothesized that we address the between-subject variability in patient’s physiological signals in response to pain.

We constructed a representation vector for each subject by computing the average normalized feature values in each pain level. Hence, we represented each subject by a 22×5 = 110 dimensional vector of F_s_ = [f_1,P0_, f_1,P1,_ …, f_22,P4_]. Then we implemented the *k*-means clustering algorithm on these vectors to group the subjects in terms of their physiological reactivity to pain. We evaluated the models on the balanced test set from the last 25% of samples not used in the clustering stage to make a fair comparison between subject-independent and hybrid clustering-based models. We explored each signal separately to find which signals can benefit from this hybrid personalization approach. According to the results in Table 8, while the EDA signals demonstrated significant improvement in one of the clusters, ECG and EMG signals could significantly benefit from the hybrid clustering-based personalization. The significance of clustering-based model improvement in each group of subjects is depicted in bold in the Table 8 and calculated by Wilcoxon signed-rank test for *p*-value < 0.05. These results imply considerable inter-subject variabilities in ECG and EMG responses to pain, which affects the automated models’ ability to generalize across people. Finding a subgroup of persons with similar ECG and EMG patterns can boost the automated pain intensity prediction models. On the other hand, EDA signal seems to be more comparable across different subjects, giving it a competitive advantage over ECG and EMG for generalized automated pain assessment model without extra effort for personalization.

**Table 8:** Comparing hybrid clustering-based and generic pain estimation model performance.

*Sensor*	*Subject group*	*Subject-independent SVR*		*Hybrid Cluster-specific SVR*	
		*MAE*	*RMSE*	*MAE*	*RMSE*
EDA	Cluster 1 (n = 14)	0.82 ± 0.15	1.04 ± 0.16	**0.80 ± 0.16**	1.03 ± 0.17
	Cluster 2 (n = 22)	0.91 ± 0.16	1.12 ± 0.18	**0.90 ± 0.18**	1.11 ± 0.20
	Cluster 3 (n = 22)	0.90 ± 0.21	1.11 ± 0.22	0.91 ± 0.20	1.12 ± 0.21
	Cluster 4 (n = 29)	0.97 ± 0.20	1.19 ± 0.22	0.96 ± 0.20	1.18 ± 0.22
	All (n = 87)	0.91 ± 0.19	1.13 ± 0.21	0.91 ± 0.20	1.12 ± 0.21
ECG	Cluster 1 (n = 16)	1.16 ± 0.19	1.34 ± 0.19	1.11 ± 0.16	1.32 ± 0.16
	Cluster 2 (n = 36)	1.19 ± 0.14	1.41 ± 0.15	**1.16 ± 0.14**	**1.37± 0.14**
	Cluster 3 (n = 7)	1.18 ± 0.15	1.39 ± 0.17	**1.13 ± 0.16**	**1.33 ± 0.16**
	Cluster 4 (n = 28)	1.14 ± 0.17	1.34 ± 0.19	**1.11 ± 0.16**	1.33± 0.17
	All (n = 87)	1.16 ± 0.16	1.37 ± 0.18	**1.13 ± 0.15**	**1.34 ± 0.16**
EMG	Cluster 1 (n = 20)	1.22 ± 0.10	1.43 ± 0.11	**1.19 ± 0.10**	1.41± 0.10
	Cluster 2 (n = 21)	1.23 ± 0.05	1.44 ± 0.05	1.22 ± 0.06	1.44 ± 0.05
	Cluster 3 (n = 18)	1.20 ± 0.11	1.41 ± 0.11	1.21 ± 0.12	1.44 ± 0.12
	Cluster 4 (n = 28)	1.22 ± 0.08	1.43 ± 0.08	**1.18 ± 0.11**	**1.40 ± 0.12**
	All (n = 87)	1.22 ± 0.09	1.43 ± 0.09	**1.20 ± 0.10**	1.42 ± 0.10

## Conclusion

In this work, we investigate the effectiveness of physiological signals for automatic pain intensity estimationthat can substitute or complement patients’ self-reported information. We explored different physiological sensors, different time-series features, and different predictive models. We explored many sensor-feature-model combinations to find the best sensor, the best subset of features, and the best machine learning model for continuous pain estimation. We evaluated the performance of the models using each individual sensor signals and combination of multiple sensor-signals. The results indicate that for both subject-independent and subject-dependent scenarios, EDA signal is the best signal for pain intensity estimation. It gave MAE = 0.93 using only 3 simple time-series features, namely, time interval between successive extreme events above the mean, time intervals between successive extreme events below the mean, and exponential fit to successive distances in 2-dimensional embedding space. We explored different machine learning models: Linear Regression, SVR, Neural Networks, KNN, Random Forest and XGBoost. In general, among all the models, SVR gave the best predictive performance across different sensors and features. We evaluated both the subject-independent and subject-dependent models and tried hybrid cluster-specific model that combines the advantages of subject-independent and subject-dependent models. In this hybrid approach, we built clustering-based-SVR models to address the inter-subject variabilities in physiological responses to pain, without need to build a new model for each subject individually. We found that ECG and EMG signals could significantly benefit from this hybrid approach, while EDA signal turns out to be a superior candidate for generic pain assessment models.

To the best of our knowledge, this is the first study that achieved less than 1-unit error for continuous pain intensity estimation using only one physiological sensor, that is EDA, 3 times series feature, and a simple SVR model. Considering that this is an encouraging result, we can estimate objective pain using only the EDA sensor which needs neither a complex setup nor a complex computationally intense machine learning algorithm. This study paves the way for developing a smart pain measurement wearable device for online pain monitoring that can change the quality of pain management significantly.

For future work, we plan to investigate the pain assessment task on data collected through cold pain experiments. One of the limitations in automated pain assessment research is that only a few datasets are available, and the proposed models and findings cannot be validated based on different scenarios. It must be noted that the pre-defined heat pain window in the BioVid dataset is 5.5 seconds, and this short window of heat pain application may not be sufficiently long for collecting physiological signals long enough for accurate automated pain intensity estimation. We will build a new pain dataset with other types of pain, different window lengths, and new and established signals. Moreover, we will explore different fusion methods to understand the benefits of different modalities while considering their practicality for a real-time pain monitoring device. There are also other challenges that remained largely unexplored in the pain literature. The physiological signals involved in responses to pain can also be affected by emotion, anxiety, sleep, and other experiences. Hence, it is essential to have a model that can distinguish the reason for these changes in physiological signals appropriately. Moreover, physiological responses to pain can be different in healthy subjects and patients suffering from specific diseases. Designing a model which can be carefully calibrated based on the different group of patients is important.
